# A New Cuffless Device for Measuring Blood Pressure: A Real-Life Validation Study

**DOI:** 10.2196/jmir.5414

**Published:** 2016-05-05

**Authors:** Tessa S Schoot, Mariska Weenk, Tom H van de Belt, Lucien JLPG Engelen, Harry van Goor, Sebastian JH Bredie

**Affiliations:** ^1^ Radboud University Medical Center Department of Internal Medicine Radboud University Nijmegen Netherlands; ^2^ Radboud University Medical Center Department of Surgery Radboud University Nijmegen Netherlands; ^3^ Radboud REshape Innovation Center Radboud University Medical Center Radboud University Nijmegen Netherlands; ^4^ Radboud University Medical Center Department of Internal Medicine and Radboud REshape Innovation Center Radboud University Nijmegen Netherlands

**Keywords:** hypertension, cuffless blood pressure monitor, wearable device, cardiovascular risk management, patient empowerment

## Abstract

**Background:**

Cuffless blood pressure (BP) monitoring devices, based on pulse transit time, are being developed as an easy-to-use, more convenient, fast, and relatively cheap alternative to conventional BP measuring devices based on cuff occlusion. Thereby they may provide a great alternative to BP self-measurement.

**Objective:**

The objective of our study was to evaluate the performance of the first release of the Checkme Health Monitor (Viatom Technology), a cuffless BP monitor, in a real-life setting. Furthermore, we wanted to investigate whether the posture of the volunteer and the position of the device relative to the heart level would influence its outcomes.

**Methods:**

Study volunteers fell into 3 BP ranges: high (>160 mmHg), normal (130–160 mmHg), and low (<130 mmHg). All requirements for test environment, observer qualification, volunteer recruitment, and BP measurements were met according to the European Society of Hypertension International Protocol (ESH-IP) for the validation of BP measurement devices. After calibrating the Checkme device, we measured systolic BP with Checkme and a validated, oscillometric reference BP monitor (RM). Measurements were performed in randomized order both in supine and in sitting position, and with Checkme at and above heart level.

**Results:**

We recruited 52 volunteers, of whom we excluded 15 (12 due to calibration failure with Checkme, 3 due to a variety of reasons). The remaining 37 volunteers were divided into low (n=14), medium (n=13), and high (n=10) BP ranges. There were 18 men and 19 women, with a mean age of 54.1 (SD 14.5) years, and mean recruitment systolic BP of 141.7 (SD 24.7) mmHg. BP results obtained by RM and Checkme correlated well. In the supine position, the difference between the RM and Checkme was >5 mmHg in 17 of 37 volunteers (46%), of whom 9 of 37 (24%) had a difference >10 mmHg and 5 of 37 (14%) had a difference >15 mmHg.

**Conclusions:**

BP obtained with Checkme correlated well with RM BP, particularly in the position (supine) in which the device was calibrated. These preliminary results are promising for conducting further research on cuffless BP measurement in the clinical and outpatient settings.

## Introduction

Noninvasive blood pressure (BP) monitors based on cuff occlusion are used widely in and outside of care facilities. These devices measure systolic (SBP) and diastolic blood pressure (DBP) by auscultation [1] or oscillometry [2]. Disadvantages of these measurements are discomfort for the patient because of painful cuff inflation, which may influence BP outcome, and the impossibility of continuous or semicontinuous BP monitoring due to the necessity of cuff inflation and deflation. Measurements can also vary between users, for example, patients or health care workers, due to interindividual differences in use. Although self-measurement of BP using noninvasive BP monitors has been shown to produce significantly greater BP reduction in patients with hypertension than standard care using clinic-based BP measurements [3], it is not common practice because it is time consuming and has high overall costs because of expensive equipment and technologies [4].

To overcome the disadvantages of BP measurements based on cuff occlusion and to provide easy-to-use devices for reliable self-measurement, pocket-sized BP monitoring devices without the need of a pressure cuff have been developed and are entering the consumer market. The majority of the cuffless devices indirectly measure BP by determining pulse transit time, the time interval required for a pressure wave in the arterial tree to travel between 2 sites (ie, a proximal and a distal point). Pulse transit time is closely related to BP via arterial compliance. Not only are these devices able to measure BP quickly and conveniently, but some of them also measure other modalities such as pulse rate, oxygenation, respiratory rate, and skin temperature. Furthermore, with respect to BP measurement, correct cuff size and cuff position are no longer important issues to take into account for obtaining reliable results. Altogether, these new cuffless devices could be an excellent alternative to BP measuring devices based on cuff occlusion, especially for the purpose of self-measurement.

The Checkme Health Monitor (Viatom Technology, Shenzen, People’s Republic of China) is a newly released Conformité Européenne-approved cuffless BP monitoring device. Checkme is a IIa category medical device compliant with directive 93/42/European Economic Community. As it is aimed at the consumer market, it has been defined as a screening device for primary medical checking and not for diagnostic use. However, for its use in a clinical setting, especially during monitoring of hypertension treatment, the device’s accuracy in persons with BPs outside the normal range has to be determined as well.

To ensure the accuracy of new BP monitoring devices, several protocols have been established, such as the European Society of Hypertension International Protocol (ESH-IP) revision 2010 [5] and protocols of the Association for the Advancement of Medical Instrumentation [6,7]. However, a single unified protocol for all types of BP monitoring devices is still under development. For example, the ESH-IP and Association for the Advancement of Medical Instrumentation protocols stipulate the use of a mercury sphygmomanometer as the reference device, whereas the International Organization for Standardization protocol allows use of any type of reference manometer, as long as it meets the accuracy requirement. Furthermore, the protocols that have been developed for validating noninvasive BP devices are designed primarily for monitors that are intrinsically able to give absolute BP readings in a single measurement.

Other category devices, such as Checkme, require patient-specific calibration by a secondary measurement method or device before they can give absolute BP readings. A protocol for validating such a monitor must include provisions to assess the monitor’s accuracy in tracking intrapatient BP changes, relative to the calibrated level, after a patient-specific calibration or between calibrations [8].

Another issue in daily practice is that oscillometric devices for the noninvasive estimation of BP have progressively become the clinical standard because of the need to train staff in determining BP by auscultation, cost, and the banning of mercury in many states and countries [2]. Therefore, it is conceivable that new devices are being evaluated in comparison with the easy-to-use automated oscillometric BP devices used in daily practice.

Finally, with classic BP devices, a correct BP can only be determined with the detection point (eg, the arm) at heart level. Because of the assumed method of BP measuring with cuffless devices, it is still unclear whether the device’s position relative to the heart may influence the results of the measurement.

The aim of this study was to evaluate the performance of the first release of the Checkme cuffless BP monitor in a real-life patient setting. To this purpose, we compared Checkme BP measurements with measurements from a validated oscillometric reference BP monitor (RM) according to ESH-IP requirements. Our second aim was to investigate whether the posture of the volunteer and the position of the device relative to the heart level would affect outcomes.

## Methods

### Checkme

**Figure 1:**
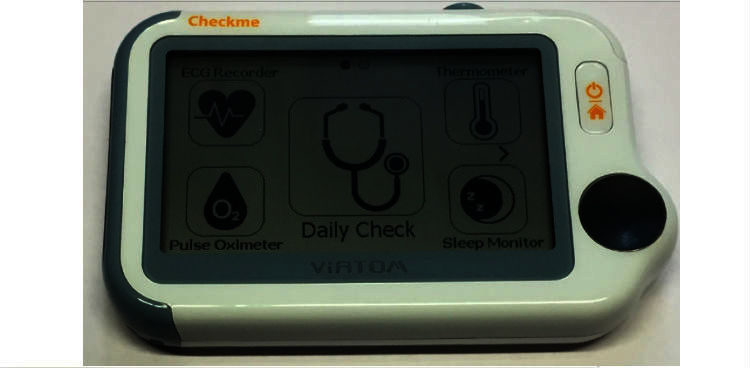
Checkme Health Monitor (Viatom Technology) device.

Checkme is a cuffless BP monitoring device, which only determines SBP. It can be used both in clinical settings and for self-measurement (Figure 1).

**Figure 2:**
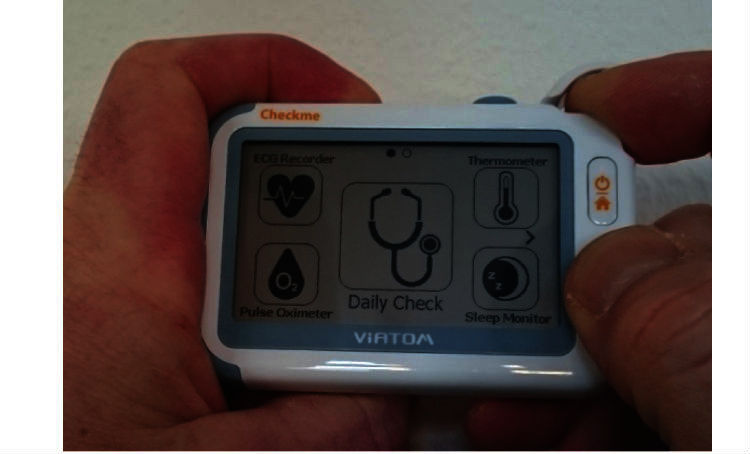
Checkme position during measurement (front).

**Figure 3:**
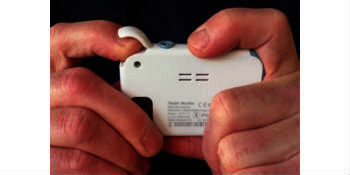
Checkme position during measurement (back).

This biometrical device can also measure skin temperature, heart rate, oxygen saturation, and 1-lead electrocardiogram, and it can be used as a sleep monitor. Before being able to measure SBP with Checkme, a personal profile containing sex, age, weight, and height has to be created, and the device has to be calibrated with an RM. Calibration is performed by simultaneously measuring SBP with Checkme and with RM and entering the SBP of the RM into Checkme after each measurement. After both calibration measurements, the Checkme is ready to use. SBP, heart rate, and oxygen saturation can then be measured by putting the right index finger beneath the lid on top, the right thumb on the metal plate on front, and the right middle finger on the metal plate on the back. Simultaneously, the metal plate on the left side of the device has to be pressed against the palm of the left hand (Figure 2,Figure 3).

**Figure 4:**
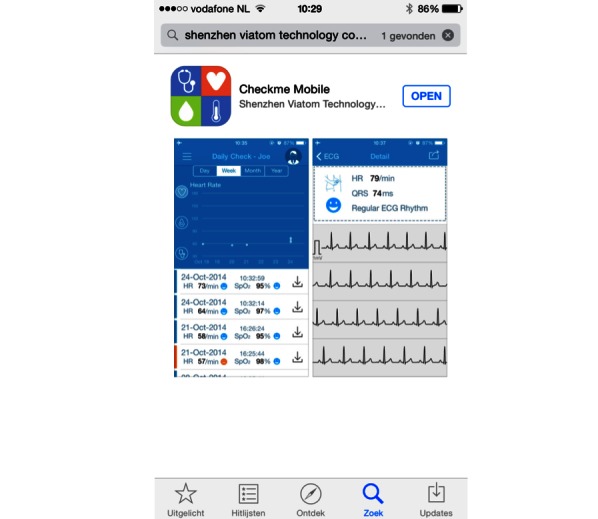
The Checkme app, showing heart rate (HR), electrocardiogram (EGC), and oxygen saturation (SpO_2_).

Checkme has to be held still at heart level during a measurement. Performing one measurement takes about 20 seconds. To evaluate the result, data can be transferred via Bluetooth to a mobile phone or tablet (supported operating systems are iOS or Android) with the Checkme app (Figure 4). Details by which the Checkme measures BP have not been described in the public domain.

### Reference Device

We used the validated Vital Signs Monitor 300 series (Welch Allyn, Skaneateles Falls, NY, USA) as RM. This automatic device measures SBP and DBP in the upper arm by oscillometry. The normal adult cuff size is suitable for people with an arm circumference of 25.3–34.4 cm. We used the small adult cuff when arm circumference was lower (range 20.0–27.0 cm) and the large adult cuff when arm circumference was higher (range 40.7–55.0 cm).

### Familiarization

Before the validation procedure, we took a multiple series of test measurements using the Checkme and RM to familiarize ourselves with the devices. To test the study procedure and familiarize ourselves with it, we measured 2 volunteers accordingly. We encountered no problems. Experienced technicians of the Radboud University Medical Center maintained and calibrated the RM according to the manufacturer’s protocol.

### Recruitment

We recruited study volunteers from patients who visited the hypertension outpatient services of the Radboud University Medical Center Department of Internal Medicine. To cover inclusion in all BP categories in this study, we also recruited patients with hypertension admitted to the hospital (highest BP range) and healthy employees (lowest BP range). We stopped recruitment after obtaining valid measurements of 37 volunteers with baseline BP measurements in the required ranges. Exclusion criteria were cardiac arrhythmias, upper-arm circumference outside the cuff range, and age <25 years. Information on age, sex, and use of antihypertension medication was collected and height, body weight, and arm circumference were measured. All volunteers gave written informed consent. The institutional review board gave permission for this study (Medical Research Ethics Committee CMO no. 2015-1717).

### Protocol

This study followed the ESH-IP requirements for test environment, observer qualification, volunteer recruitment, and BP measurements for the validation of BP measurement devices [5]. Because device readings are digital, 1 researcher performed all measurements. In addition to the ESH-IP requirements, we took measurements in different positions to establish the influence of posture on device readings.

Each volunteer was seen individually in a quiet, temperature-controlled room. Appropriate cuff size (in the case of RM) was chosen based on upper-arm circumference. For each individual volunteer, a new profile was created on the Checkme device, with input of sex, date of birth, height, and weight. Volunteers were given oral instructions regarding proper use of the Checkme device before measurements were taken.

Baseline measurements were performed with the volunteer in the supine position after resting for 10 minutes. BP was measured 3 times at the right upper arm with the RM. The mean of the last 2 values was used as the baseline value, on the basis of which volunteers were divided into 1 of 3 BP categories: high (SBP >160 mmHg), normal (SBP ≥130 and ≤160 mmHg), or low (SBP <130 mmHg) BP, according to ESH-IP, with at least 10 volunteers in each BP category.

Next, we calibrated the Checkme device with the volunteer in the supine position with hands resting on the lower abdomen. The last measured baseline SBP with the RM was used as the input value for calibration. After calibration, we randomized the order of measurements. In the first series of measurements, BP was measured in the supine position with Checkme at heart level (arms resting on lower abdomen), Checkme above heart level (arms stretched above the head at a 90° angle with the body), and the RM (right upper arm) according to the randomization order. After the first series of measurements in the supine position, volunteers were asked to sit up. After 5 minutes of rest, the volunteer’s BP was again measured in random order with the Checkme at heart level and RM, both in the upright position. All of the above measurements were executed 3 times successively. According to ESH-IP, the interval between consecutive measurements was between 30 and 60 seconds. Failed measurements were repeated up to a maximum of 3 times.

### Statistical Analysis

All statistic calculations were performed with IBM SPSS version 20 (IBM Corporation). To evaluate the influence of the volunteer’s position on the device readings, we compared the means of 3 consecutive measurements with a device in the supine or sitting position by paired samples *t* -test. A difference with *P*<.05 was considered to be significant.

## Results

We excluded 15 of 52 recruited volunteers: 12 due to repeated BP calibration failures with Checkme, 2 because they appeared to have low BP (SBP <130 mmHg) with already sufficient data, and 1 who declined to continue after inclusion. None of the volunteers had arrhythmias. In <3% of all measurements, BP had to be measured again due to failure during the first attempt of both the RM and the Checkme readings.

### Study Population

Of the 37 volunteers who completed the study, 14 were in the low range (SBP <130 mmHg), 13 were in the medium range (SBP between 130 and 160 mmHg), and 10 were in the high range (SBP >160 mmHg). Table 1 shows their baseline characteristics. There were 18 men and 19 women with a mean age of 54.1 (SD 14.5) years. The mean baseline SBP was 141.7 (SD 24.7) mmHg. For 31 of the 37 volunteers (84%) we used the normal cuff size of the RM. Due to an arm circumference above than normal range, the remaining 6 volunteers (16%) required the large cuff.

### Feasibility

In 22 of 52 volunteers (42%), calibration with Checkme failed the first time (error message: “unstable measure, calibration failed”). We repeated the procedure up to a maximum of 5 times. In 5 of 52 volunteers (10%), calibration succeeded after the second attempt, in 4 (8%) after the third attempt, and in 1 (2%) after the fifth attempt. Calibration continued to fail in 12 of 52 volunteers (23%), whereupon they were excluded from further measurements. In 2 of 37 volunteers who completed the study, the SBP measurement could not be determined in the upright position.

### Comparing BP Results (Primary Aim)

**Figure 5:**
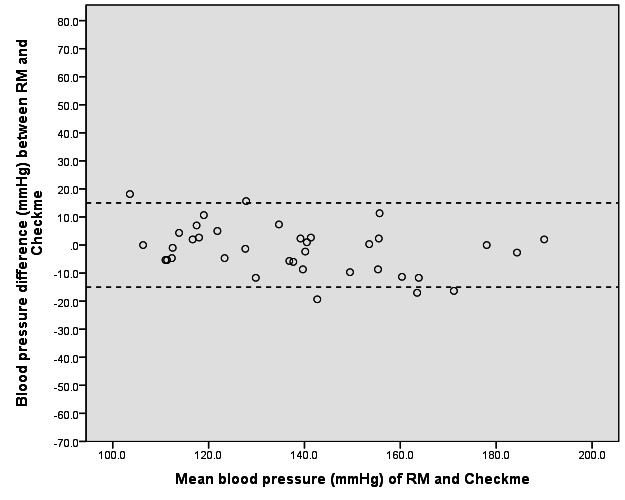
Bland-Altman plot of the difference in systolic blood pressure readings between the reference monitor (RM) and the Checkme Health Monitor (at heart level) in the supine position.

**Figure 6:**
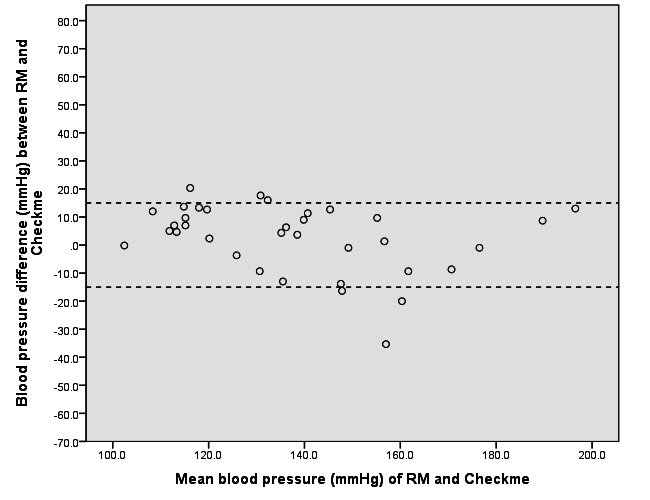
Bland-Altman plot of the difference in systolic blood pressure readings between the reference monitor (RM) and the Checkme Health Monitor (at heart level) in the upright position.

Table 2 shows the BP results for RM and Checkme. Table 3 shows the proportion of patients with differences between RM and Checkme of >5, >10, and >mmHg. We constructed Bland-Altman scatter plots of BP differences between RM and Checkme against the mean BP of the RM and Checkme in the supine (Figure 5) and upright positions (Figure 6). BP results correlated with the position of Checkme relative to the heart level.

**Table 1:** Study population characteristics.

Characteristics	All volunteers (n=37)
Male:female	18:19
Age in years, mean (SD)	54.1 (14.5)
White, n (%)	36 (97)
Black, n (%)	1 (3)
Height in m, mean (SD)	172.2 (7.5)
Weight in kg, mean (SD)	83.3 (18.4)
Use of blood pressure-lowering drugs, n (%)	22 (60)
Normal cuff size, n (%)	31 (84)
Baseline systolic blood pressure in mmHg, mean (SD)	141.7 (24.7)

**Table 2:** Systolic blood pressure measurements (mmHg) taken by the reference monitor and Checkme in the supine and upright positions.

Volunteers’ position		Mean^a^	SD	Range (min; max)^a^
**Supine position**				
	Reference monitor	136.6	21.8	84.7 (106.3; 191.0)
	Checkme at heart level	138.4	25.2	94.5 (94.5; 189.0)
	Checkme above heart	130.7^b^	27.7	101.0 (86.0; 187.0)
**Upright position**				
	Reference monitor	139.2	22.3	100.7 (102.3; 203.0)
	Checkme at heart level	136.6^c^	25.9	87.7 (102.3; 190.0)

^a^The average or range of 3 consecutive blood pressure measurements.

^b^ *P*<.001 compared with Checkme at heart level.

^c^ *P*=.01 compared with Checkme at heart level in the supine position.

**Table 3:** Differences in systolic blood pressure readings between the reference monitor and Checkme in various postures and the proportion of volunteers with differences >5, >10, and >15 mmHg between the reference monitor and Checkme.

Reading differences		Supine at heart level (n=37)	Upright at heart level (n=35)
**Difference between the devices (mm Hg)**			
	Mean (SD)	–1.8 (8.5)	2.6 (12.1)^a^
	Min; max of range	–19.3; 18.2	–35.5; 20.3
**Degree of difference**			
	>5 mmHg, n (%)	17 (46)	23 (66)
	>10 mmHg, n (%)	9 (24)	15 (43)
	>15 mmHg, n (%)	5 (14)	6 (17)

^a^ *P*=.02 compared with measurements in the supine position.

### Influence of Posture on the Device Readings (Secondary Aim)

Table 2 summarizes the results of the SBP measurements obtained with RM and Checkme in the various positions. In the supine position, SBP measured with Checkme above heart level was significantly lower than SBP measured supine at heart level. SBP obtained with Checkme in the upright position was significantly lower than in the supine position, in which the device is just above heart level. Table 3 summarizes differences in SBP readings between RM and Checkme in both the supine and upright positions. The SBP measurement with Checkme in the upright position was significantly lower than the SBP measurement with Checkme in the supine position.

## Discussion

The results of this comparative study show that the first version of the Checkme device yields BP results that are to a large extend comparable with BPs obtained by a validated oscillometric BP monitor. We observed this for a predefined wide range of BP levels under well-controlled circumstances. Furthermore, BP results correlated with the position of Checkme relative to the heart level. Compared with a reference BP, Checkme recorded a higher BP below heart level and a lower BP above heart level.

Due to the lack of a uniform international protocol that includes provisions to assess intrapatient BP changes relative to the calibrated level, it was not possible to conduct a formal device validation study. As the Checkme requires patient-specific calibration by a secondary measurement device before it can measure absolute BP, we consider such a protocol to be necessary.

The strength of this study is that it met all ESH-IP requirements for test environment, observer qualification, volunteer recruitment, and BP measurements. Measurements were conducted in a quiet, temperature-controlled room and the manufacturer’s guidelines on use of the test device were followed. Furthermore, we used a validated RM device and randomized the order of measurements with Checkme and RM to eliminate the influence of changes in BP over time on the study results.

Checkme is one of the first cuffless devices to be launched, indicating that cuffless BP measurement is in its infancy. Notably, Checkme has outgrown its developmental phase. As the technique of cuffless devices is continuously being improved, future generations of Checkme may be even more suitable for measuring BP in the clinic.

One disadvantage of Checkme is the inability to measure DBP, because DBP can be used to calculate pulse pressure and adds to the overall cardiovascular risk profile. Based on the underlying method of measuring, a subsequent version of Checkme may expected to have this ability. Another issue with the Checkme version used in this study was the inability to calibrate the device in a substantial number of volunteers. Repeated attempts to calibrate Checkme after warming volunteers’ hands and further instructing them to hold still or change their position were not effective in some of them and thus further BP measurements were not possible. According to the manufacturer, a new software release has resolved this problem.

Ideally, Checkme is calibrated by taking simultaneous BP measurement with the RM. In this study, we calibrated Checkme after baseline measurements with the RM. However, as the time interval between taking the 2 measurements was a maximum 2 minutes (depending on the number of attempts during calibration), we can assumed that BP had not significantly altered. Calibration parameter stability over longer periods of time has yet to be established in further research. After the completion of this study, Viatom updated the Checkme software to reduce calibration failures and has provided additional instructions for positioning Checkme against a lower limb during the calibration measurements. Therefore, the process of calibration can be expected to be more successful in future studies.

Checkme’s BP measuring algorithm has not been made public, probably for commercial reasons. Most cuffless devices measure BP indirectly by determining pulse transit time, the time interval required for a pressure wave in the arterial tree to travel between 2 sites (ie, a proximal and a distal point). Pulse transit time is closely related to BP via arterial compliance. For example, if arterial BP increases, arterial wall tension will increase. Subsequently, arterial compliance and pulse transit time will decrease [9]. Most cuffless devices calculate pulse transit time by using the electrocardiogram as the proximal timing reference and the arterial waveform in an extremity as the distal reference [10]. Recent research has shown a significant relationship between BP measured with pulse transit time and BP measured with conventional devices based on cuff occlusion [11-13].

Differences in BP depending on posture and position of the device suggest that cuffless BP measurement by Checkme, and probably in general, is influenced by the position of the device relative to heart level. This may suggest an inherent error in Checkme’s algorithm when BP is measured in a position other than that indicated by the manufacturer. Therefore, it is important that future users of Checkme conduct all measurements in the position stipulated in the user manual. Furthermore, we observed 1 outlier (with SBP difference between RM and Checkme >40 mmHg), which we could explain.

If Checkme will be able to fulfill formal international validation protocol requirements, which include provisions to assess the monitor’s accuracy in tracking intrapatient BP changes relative to the calibrated level, after a patient-specific calibration or between calibrations, we expect increased use of this device. Especially promising is such devices’ ability to measure BP faster and more conveniently than conventional BP monitoring devices based on cuff occlusion. This implies not only that BP can be measured more efficiently in the clinic, but also that patients can easily self-monitor their BP at home. Because self-measurement of BP has been shown to have a positive effect on reducing BP [3], this easy-to-use BP device will probably find a place in the management of hypertension. The low costs of cuffless devices relative to cuff occlusion devices will also contribute to their implementation in and outside the clinic.

We believe the market of wearable BP sensors will develop in the areas of self-measurement and remote monitoring. In this context, device validation may be accelerated if development of techniques, calculation, and feedback on the basis of clinical data would take place in an open source environment.

### Conclusion

Checkme SBP correlated well with reference SBP, in particular in the supine position. Although we did not perform a formal validation study at this preliminary stage, these preliminary results are most promising and warrant further research on cuffless BP measurement in the hospital, the clinic, and at home.

## Abbreviations

BP: blood pressure
DBP: diastolic blood pressure
ESH-IP: European Society of Hypertension International Protocol
RM: reference blood pressure monitor
SBP: systolic blood pressure
